# Documenting good practices: scaling up the youth friendly health service model in Colombia

**DOI:** 10.1186/s12978-015-0079-7

**Published:** 2015-09-18

**Authors:** Silvia Huaynoca, Joar Svanemyr, Venkatraman C. Chandra-Mouli, Diva Jeaneth Moreno Lopez

**Affiliations:** Independent Consultant, 125 Maiden Lane, 9th Floor, New York, NY 10038 USA; Department for Reproductive Health and Research, WHO, 20 Avenue Appia, 1211 Geneva, Switzerland; Dirección de Promoción y Prevención, Ministerio de Salud y Protección Social Colombia, 37-76 1st Floor, Carrera 13, PO Box 110311, Bogotá, Colombia

## Abstract

**Background:**

Young people make up for 24.5 % of Latin America’s population. Inadequate supply of specific and timely sexual and reproductive health (SRH) services and sexuality education for young people increases their risk of sexual and reproductive ill health. Colombia is one of the few countries in Latin America that has implemented and scaled up specific and differentiated health and SRH services-termed as its Youth Friendly Health Services (YFHS) Model.

**Objective:**

To provide a systematic description of the crucial factors that facilitated and hindered the scale up process of the YFHS Model in Colombia.

**Methods:**

A comprehensive literature search on SRH services for young people and national efforts to improve their quality of care in Colombia and neighbouring countries was carried out along with interviews with a selection of key stakeholders. The information gathered was analysed using the World Health Organization-ExpandNet framework (WHO-ExpandNet).

**Results/Discussion:**

In 7 years (2007–2013) of the implementation of the YFHS Model in Colombia more than 800 clinics nationally have been made youth friendly. By 2013, 536 municipalities in 32 departments had YFHS, resulting in coverage of 52 % of municipalities offering YHFS.

The analysis using the WHO-ExpandNet framework identified five elements that enabled the scale up process: Clear policies and implementation guidelines on YFHS, clear attributes of the user organization and resource team, establishment and implementation of an inter-sectoral and interagency strategy, identification of and support to stakeholders and advocates of YFHS, and solid monitoring and evaluation.

The elements that limited or slowed down the scale up effort were: Insufficient number of health personnel trained in youth health and SRH, a high turnover of health personnel, a decentralized health security system, inadequate supply of financial and human resources, and negative perceptions among community members about providing SRH information and services to young people.

**Conclusion:**

Colombia’s experience shows that for large-scale implementation of youth health programmes, clear policies and implementation guidelines, support from institutional leaders and authorities who become champions of YFHS, continuous training of health personnel, and inclusion of users in the design and monitoring of these services are key.

## Abstract (es)

**Abstract:**

En América Latina los jóvenes constituyen el 24.5 % de la población total. La inadecuada e inoportuna oferta de servicios de salud sexual y reproductiva (SSR) y educación sexual para jóvenes incrementa su riesgo a una inadecuada SSR. Colombia es uno de los pocos países en América Latina que ha implementado a nivel nacional servicios de SSR específicos y diferenciados para jóvenes -conocidos como Modelo de Servicios de Salud Amigables para Adolescentes y Jóvenes (SSAAJ).

**Objetivos:**

Proporcionar una descripción sistemática de los factores cruciales que facilitaron y obstaculizaron la ampliación a escala del Modelo SSAAJ en Colombia.

**Metodología:**

Basada en una revisión bibliográfica sobre servicios de SSR para jóvenes, esfuerzos nacionales para mejorar la atención de calidad en Colombia y países vecinos y entrevistas a aliados clave. La información recolectada fue analizada con el marco ExpandNet de la Organización Mundial de la Salud.

**Resultados/Discusión:**

En los siete años (2007–2013) de implementación del Modelo SSAAJ mas de 800 clínicas a nivel nacional se convirtieron en amigables para jóvenes. En el 2013, 536 municipalidades en 32 departamentos tenían SSAAJ alcanzando una cobertura de 52 % por municipalidad.

El análisis de la información identificó cinco elementos que facilitaron la ampliación a escala: políticas y guías claras de implementación de SSAAJ, atributos claros de la organización usuaria y del equipo de recursos, establecimiento e implementación de una estrategia interinstitucional e intersectorial, identificación de aliados y abogadores de los SSAAJ, y sólidos monitoreo y evaluación.

Los elementos que limitaron o retrasaron este esfuerzo fueron: insuficiente número del personal de salud capacitado en salud del joven y SSR, alta rotación del personal de salud, descentralizado sistema de seguro de salud, inadecuada dotación de recursos financieros y humanos, y percepciones negativas de miembros comunitarios sobre la oferta de información y acceso a servicios de SSR para jóvenes.

**Conclusión:**

La experiencia en Colombia muestra que en la ampliación a escala de programas de salud para jóvenes son esenciales políticas de salud con claras directrices de implementación, apoyo de líderes y directivos institucionales trabajando en SSAAJ, formación continua del personal de salud e inclusión de usuarios en el diseño y supervisión de servicios.

**Palabras clave:**

Servicios de salud amigables, salud sexual y reproductiva, ampliación a escala.

## Introduction

Young people^1^ make up 27 % of the world’s population [1, 2] and 24.5 % of Latin America’s (LA) population [3]. Despite being considered a healthy group, young people are at higher risk of sexual and reproductive morbidity and mortality [4–7] and more likely to experience difficulties in obtaining specific and timely sexual and reproductive health (SRH) services and sexuality education, limiting their ability to realize SRH benefits [8–11].

During the last few years a large number of countries have expressed a commitment to providing young people SRH education and services. However, in only a small number of countries have SRH initiatives and interventions moved from small scale and time limited projects to comprehensive, large scale and sustained programmes [12–14]. Colombia is one of the few countries in Latin America that has been able to implement and scale up differentiated SRH services, better known as Youth Friendly Health Services (YFHS) (in Spanish, Servicios de Salud Amigable para Adolescentes y Jóvenes) [15]. By 2013 850 YFHS had been established across the country [16] .

The SRH of young Colombians has been a national public health concern and priority. In the past 20 years the proportion of pregnant adolescents aged 15–19 increased significantly (from 13 % in 1990 to 20.5 % in 2005) [17]. In the same age group there was an increase in the fertility rate (from 70 to 90 births per 1000 women between 1990 and 2005) [17] and in the unmet need and demand for family planning (from 10.7 to 14.4 % and from 93.9 to 96.6 % respectively); contraceptive use remained with an insignificant decrease from 83.2 % in 2000 to 82.2 % in 2010 [18]. Young women living in conditions of vulnerability - coming from rural areas and with low levels of education and economic income, are at higher risk of early pregnancies [19].

In 2007 the Colombian government initiated the implementation of YFHS in 10 departments of the country, in the context of a project supported by UNFPA [20, 21]. Since the inception of these services the MHSP had in mind a national implementation. Nevertheless, it did not have a clear planned strategy to do so but a national law that mandated its fulfilment.

This article presents and analyses the key factors and attributes that facilitated the scale up of YFHS in Colombia. The first part provides a description and a historical overview of the YFHS model implemented in Colombia. The second part presents an analysis of the scale up process using the ExpandNet framework of the World Health Organization (WHO-ExpandNet). The introduction of YFHS has not be completely smooth and some of the challenges and hinders met are also described and discussed. Finally, the article singles out some lessons learned and recommendations that can be useful in different contexts.

## Methodology

### Data Sources

We conducted a systematic and comprehensive literature search and review of SRH services for young people as well as national efforts to improve their quality of care targeting Colombia and neighbouring countries. We covered published and unpublished literature in Spanish and English.

The systematic search included various combinations of the following concepts: “Colombia”, “Latin America”, “adolescents”,”youth”,“friendly health services”,“health services” and “reproductive health” in 8 databases (MEDLINE, PubMed, SCiELO, Academic Search Complete, CINAHL plus, Cochrane library, HAPI online, LILACS REPIDISIC) and 6 websites of international organizations (WHOLIS, Pan American Health Organization, UNFPA, UNICEF, UNESCO, Andean Plan to Prevent Adolescent Pregnancy, and World Bank e-Library).

In the initial search we identified 430 articles, of which we selected 86 for initial review. Based on our inclusion and exclusion criteria, we identified one article and 11 reports on the YFHS Model in Colombia and six scientific articles and 14 reports on YFHS in LA for the final review (Table 1).

**Table 1:** Publications on youth friendly health services in Colombia

Title of the publication	Source or Author(s)/Year
“Este es tu centro, socio” Una experiencia exitosa de servicios de salud amigables para jóvenes en Bogotá, Colombia. “This is your partner center” A successful experience of adolescent friendly health services in Bogota, Colombia.	Toloza-Pérez; 2013
Servicios amigables en salud para adolescentes y jóvenes. Un modelo para adecuar las respuestas de os servicios de salud a las necesidades de adolescentes y jóvenes de Colombia. Adolescent friendly health services. A model for adapting the responses of health services to the needs of young people in Colombia.	Colombia: MSPS, UNFPA; 2007
Lecciones aprendidas del proyecto del Fondo Mundial en Colombia. Proyecto Colombia. Lessons learned from the Global Fund project in Colombia. Project Colombia.	Fernández D; 2007
Servicios amigables para jóvenes: Construcción conjunta entre jóvenes y funcionarios. Health care services for young people: A joint construction among youth and health personnel.	Valencia CP, et. Al; 2010
La implementación de servicios de salud amigables para adolescentes y jóvenes en el departamento de Huila. La experiencia de las ESE de Campoalegre, La Plata y Neiva. Implementing adolescent friendly health services in the department of Huila. Experience in Campoalegre, La Plata and Neiva.	Colombia: MSPS; 2010
Intercambio de experiencias internacionales: Modelo SSAAJ. Exchange of international experiences: Adolescent Friendly Health Service Model.	Plan Andino Prevención Embarazo Adolescente; 2011
Avances en la implementación del modelo de servicios de salud amigables para adolescentes y jóvenes. Análisis y evaluación 2010. Progress in implementing the adolescent friendly health services model. Analysis and Evaluation.	Colombia: MSPS, UNFPA; 2011
Evaluación sumativa de los Servicios de Salud Amigables para Adolescentes y Jóvenes en Colombia. Summative Assessment of Adolescent Friendly Health Services in Colombia.	Colombia: MSPS, UNFPA; 2011
Compromiso de los partidos y movimientos políticos. Por la promoción y la garantía de los derechos humanos, sexuales y reproductivos. Commitment of political parties and movements. For the promotion and guarantee of human, sexual and reproductive rights.	Colombia: UNFPA; 2011
Indicadores de productividad de los SSAAJ y reporte de las Direcciones Territoriales de Salud, Productivity of AFHS indicators and Regional Directorate of Health reporting.	Colombia: MSPS; 2012
Servicios de salud amigables para adolescentes. Una revisión de su implementación y principales características. Adolescent Friendly Health Services. A review of its implementation and main characteristics.	Moreno-López D, Púa-Mora R; 2012
Informe de Actividades 2012–2013. Sector Administrativo de Salud y Protección Social al Honorable Congreso de la República. Activity Report 2012–2013. From the Health and Social Protection Administrative Sector to the Congress of the Republic.	Colombia: MSPS; 2013

The inclusion criteria was that publications should present findings regarding the implementation and scale up of YFHS Model in Colombia and the status of YFHS and/or the SRH of young people in Colombia and LA in general. We excluded articles that focused on youth centres or programmes that did not implement the YFHS Model in Colombia.

Finally we collected and reviewed plans, reports, and presentations from the Ministry of Health and Social Protection (MHSP) and the United Nations Population Fund (UNFPA) in Colombia. We also had meetings with representatives from the same agencies.

### Framework for Analysis

The WHO-ExpandNet Framework is used to define and identify actors and actions that took place during the scale up process of the YFHS Model in Colombia.

The WHO-ExpandNet framework was developed by WHO to assist countries in scaling up health interventions with the aim of reaching more people, more rapidly and in more sustainable ways [22]. The WHO-ExpandNet framework establishes a practical scaling up guide, which comprises two sections that complement one another. The first section guides the systematic planning of the scale up strategy. Its elements are: the innovation to be scaled up, the user organization, the resource team, and the environment. The second section guides the strategic management of the scale up process, which is composed by the dissemination and advocacy, the organizational process, the costs/resource mobilization, and the monitoring and evaluation. Each of these elements must meet specific requirements for implementation. Tables 2 and 3 present the definition of these elements, as well as their characteristics of good practice.

**Table 2:** World Health Organization-expandNet framework-planning the scaling up strategy

Elements	Definition	Characteristics of good practice
Innovation	The interventions and/or practices to be scaled up	Relevant, Credible, Clear, Compatible with values and norms, Easy to install
User Organization	The institution that adopts and implements the innovation at scale	Credible, Commitment, Capacity
Resource Team	Individuals and organizations that have been involved in the development and testing of the innovation and/or seek to promote its wider use	Leadership, Credibility, Commitment, Capacity
Environment	The conditions and institutions, external to the user organization, that substantially affect the prospects for scaling up	Understanding the challenges and opportunities in the environment and taking them into account
Vertical scaling up strategy	The policy, political, legal, regulatory, budgetary or other health systems changes needed to institutionalize the innovation	It ‘legitimizes’ the innovation, integrates it in national and sub-national work plans and budgets and thus increases the likelihood of it being applied nationwide over a sustained period
Horizontal scaling up strategy	The replication of the innovation in different geographic sites or its extension to larger or different population groups.	Wider application and reach out of the innovation

Source. WHO 2010. Nine steps for developing a scaling-up strategy, Geneva, Switzerland, World Health Organization

**Table 3:** World Health Organization-expandnet framework-strategic management of the scale up effort

Elements	Characteristics
Communication and advocacy	It is the availability of appropriate approaches and relationships for advocacy on, introduction of, and information about the innovation to reach key audiences
Management and organization	It is the path followed by the scale up process. It stresses the importance of charting out the management process, its pace and scope, whether it is to be centralized or decentralized, whether it is to be adaptive or fixed and who would drive the process
Resources	It stresses the importance of integrating scaling up efforts into national and sub-national work plans and budgets, and of tapping into existing funding mechanisms
Monitoring and evaluation	It stresses the critical importance of monitoring and evaluation using methods such as such as routinely gathered statistics, special surveys and formative and intervention-effectiveness research

Source. WHO 2010. Nine steps for developing a scaling-up strategy, Geneva, Switzerland, World Health Organization

The WHO-ExpandNet framework is also relevant to evaluating how scaling up of health interventions is done. In line with an approach we used in another paper [12], we decided to use this framework because it breaks down the model for scale up into pieces that can be examined closely and provides clear objective analysis criteria.

### History of introducing and scaling up the YFHS Model in Colombia

Colombia has a long history of political commitment to making SRH services available for young people. The creation and implementation of the Program of Comprehensive Care for Adolescents in 1993 [23] and the prioritization of youth SRH strategies, in both the 2003 National Policy on Sexual and Reproductive Health and the 2007–2012 National Plan of Public Health, are among the most relevant milestones [24, 25].

The implementation of differentiated services by the MHSP for young people began in the 1990’s as projects with limited geographical, temporal, and financial scopes. The effort of the Global Fund for AIDS Tuberculosis and Malaria to implement differentiated SRH services for young people in 48 municipalities of 25 departments between 2005 and 2008 provided important lessons learned to design the YFHS Model [26]. Firstly, it confirmed the perceived need of health facilities with availability of adequate SRH services and information for young people [26]. Secondly, it showed that the absence of standardized protocols to assist young people, flexible schedules of operation, and processes for monitoring services can hinder the quality and sustainability of services provided [27]. The HIV/AIDS programming stimulated scale up since it provided a broader approach to HIV including comprehensive sexuality education and availability of youth friendly services.

In 2007, the MHSP signed an agreement of collaboration with UNFPA to design and implement differentiated health services for young people, known as the YFHS Model. The design of the Model followed a process that included a review of international recommendations for making services youth friendly, meetings within the MHSP, the General Social Security System (GSSS), and UNFPA to assess the feasibility of such recommendations, and consultations with young people to identify how well these services were received and met youth needs. At the end of this process the MHSP presented the goals of the YFHS Model as to reduce barriers to accessing SRH services, to promote active participation of young people in the design of SRH programs, and to strengthen the institutional capacity of the GSSS [20].

The MHSP, the GSSS and UNFPA, based on the characteristics of the Colombian health system and its capacity to provide services, recommended that the Model followed two principles. Firstly, health facilities adopting the Model should have five components: a) easy and timely accessibility, b) personnel trained in YFHS, c) administrative and management processes in place that respond to quality standards of YFHS provision, d) availability of a wide range of health services, and e) youth, community, and social participation as well as inter-sectoral coordination (See Table 4). Secondly, the health facility would adopt one of three types of set up: a) differentiated service, b) friendly unit or c) friendly centre (See Table 5). This design was subject to review and adjustment depending on conditions and realities of the different regions in the country and the providers’ execution ability to implement the Model [20].

**Table 4:** Components and characteristics of the AFHS Model in Colombia

Component	Characteristics
Access and opportunity in service delivery	Infrastructure and geographical accessibility
	Physical setting
	Identification of the service
	Differentiated hours of operation and appointment scheduling
	Enabled services based on national policies in sexual and reproductive health
	Acknowledgment of services by adolescents and young people
Health professionals and staff	Trained in adolescent friendly and differentiated services
	Confidential
	Respectful of cultural and gender diversity, economic situation, etc.
	Capable to identify prejudices, stereotypes and emotions that make it difficult to empathize or provide services
	Not feeling obligated to abandon personal beliefs or values; but willing to understand views of adolescents
Administrative and management procedures	Suitable to provide comprehensive services
	Adoption of national standards and policies (Decree 1011 of 2006)
	Readjusted route that adolescents and young people follow from the moment they enter until they leave the facility
Availability of a wide range of services	Defined according to the set up of care and the needs of young people
	Continued with other levels of care/reference and counter-reference levels
Youth, social, and community participation as well as inter-sectorial coordination	Services empowered by young people
	Inter-sectorial actions
	Working agreements with social organizations

Source: Ministerio de Salud y Protección Social Colombia, UNFPA. (2007). Servicios amigables en salud para adolescentes y jóvenes. Un modelo para adecuar las respuestas de los servicios de salud a las necesidades de adolescentes y jóvenes de Colombia. Bogotá D.C., Colombia

**Table 5:** Set ups of the AFHS model in Colombia

Set up	Characteristics
Differentiated service	Delivery of services by a trained professional in AFHS in any health facility. Services may be available within the normal operation hours of the facility.
Friendly Unit	Physical space, in a facility, destined for young people, with differentiated hours of operation and run by trained professional in AFHS
Friendly Facility	Exclusive facility for adolescents and young people with trained professional in AFHS and with areas of interaction for young people

Source: Ministerio de Salud y Protección Social Colombia, UNFPA. (2007). Servicios amigables en salud para adolescentes y jóvenes. Un modelo para adecuar las respuestas de los servicios de salud a las necesidades de adolescentes y jóvenes de Colombia. Bogotá D.C., Colombia

The scale up of the YFHS Model followed a systematic path and assigned specific responsibilities to key players at different levels of the health system; which consists of the MHSP and the GSSS. The MHSP as the national entity in charge of developing public health policies, mandated the implementation of the Model, worked with the Regional Directorate of Health (RDH) and Local Directorate of Health (LDH) to press for the inclusion of the Model in their operational plans, allocation of financial and human resources, and incorporation of information systems to ensure the implementation and monitoring of the Model, and providing training on YFHS to the GSSS’ personnel. The GSSS as the set of programmes and institutions that directly provide health services to the population through private and public Providers of Healthcare Services (PHS), made available one of the three modalities of care of YFHS [15, 20].

The scale up of the YFHS Model followed vertical and horizontal strategies. The vertical strategy involved institutionalizing the Model within the MHSP and GSSS. Table 6 lists in chronological order the enactment of laws and resolutions that supported this scale up.

**Table 6:** Political process that strengthened the implementation of the AFHS Model in Colombia

1968	Creation of the National Institute for Youth and Sports
1990-1994	Creation of the Presidential Program for Youth, Woman and Family
1993	Launch of the Manual of Technical and Administrative Regulations for Comprehensive Care of Youth
1994–1998	Creation of the Vice Ministry of Youth, under the Ministry of Education.
	In 2000 becomes the Presidential Program Young Colombia
1997	Enactment of Act 375 or Youth Law
2003	Enactment of the National Policy on Sexual and Reproductive Health (SRH)
2004	Circular 18. Health insurance companies and local secretaries of health guarantee care for young people
2006	Act 1098. Code for Children and Adolescents. Guarantees free access to SRH services for young people, development of programs to prevent unwanted pregnancies, and priority attention to adolescent mothers
2006	Sentence C355 and Decree 4444. Decriminalize abortion in case of: a) Pregnancy endangers life/health of women, b) Severe fetal malformation, c) Pregnancy result of sexual intercourse or sexual act without consent
2007	Decree 3039. Adopts the National Plan of Public Health and joins the Plan Andino to Prevent Adolescent Pregnancy
2007	Act 1122. Recommends the implementation of the Adolescent Friendly Health Services (AFHS) Model
2008	Beginning of the national implementation of the model AFHS
2008	Resolution 425. Collective Intervention Plan. Contemplates mandatory implementation of the AFHS model
2010	Decree 2968. Creates the National Inter-sectorial Commission to promote and guarantee sexual and reproductive rights

Source: Ministerio de Salud y Protección Social Colombia, UNFPA. (2007). Servicios amigables en salud para adolescentes y jóvenes. Un modelo para adecuar las respuestas de los servicios de salud a las necesidades de adolescentes y jóvenes de Colombia. Bogotá D.C., Colombia

Mejía-Gómez ML, Montoya-Chica P, Blanco-Rojas AJ, et al. (2010). Barreras para el acceso de adolescentes y jóvenes a servicios de salud. Propuesta para su identificación y superación. Documento regional - 2010 Bogotá D.C., Colombia

Ministerio de Salud y Protección Social Colombia. (2010). La implementación de servicios de salud amigables para adolescentes y jóvenes en el departamento de Huila. La experiencia de las ESE de Campoalegre, La Plata y Neiva. Bogotá D.C

The horizontal strategy consisted of countrywide scaling up the Model in departments, districts, and municipalities. In 2007 the YFHS Model was approved to be implemented nationally; but without a clear plan in terms of coverage. In 2008 it was officially launched in ten departments. The scale up process took place in phases with an annual increase of health facilities adopting the Model. By 2009, 290 health facilities had implemented the Model. In the following four years a total of 817 new ones were added (371, 213, 49, and 184 in 2010, 2011, 2012 and 2013 respectively). The 2010 qualitative evaluation reported that the total of health facilities with the Model, 84 % of them were differentiated services, 14 % friendly units, and <1 % friendly centers [28]. By 2013, 30 out of 32 departments and 536 out of 1198 municipalities had YFHS (52 % in municipalities and 94 % in the public sector) [29]. Although these numbers mean there is still a way to go to reach national coverage this level of scale is among the most successful in Latin America.

## Results: Applying the ExpandNet framework to examine the scale up of the YFHS Model

This section is divided into two main segments: The systematic planning and the strategic management of the scale up process. In each segment, we assessed the key elements of the WHO-ExpandNet framework and whether or not they fulfil the criteria of good practice. (See Tables 2, 3).

### Planning the scaling up strategy

#### Innovation

The innovation or the intervention to be scaled up was the YFHS Model. According to the WHO-ExpandNet framework, the attributes of good practices of innovations are clarity, credibility, relevancy, compatibility, and easy installation.

In Colombia the attributes of the YFHS Model were clear. The MHSP in collaboration with UNFPA developed a manual titled ‘Friendly Health Services for Young People’ to create a shared understanding of the YFHS Model’s characteristics. The manual defined youth friendly services as “health facilities that provided care to young people aged 10–29, offered a range of accessible and timely services and quality care, and were adapted to young people’s reality [20]”. To be considered youth friendly these facilities guaranteed geographic accessibility, privacy and confidentiality, adequate hours of operation for young people, health personnel trained in YFHS, availability of a wide range of services, and opportunities for young people and members of the community to participate in the continuous improvement of these facilities.

The manual provided health personnel and authorities from the GSSS with national quality standards for the design, implementation, monitoring, and evaluation of these services. This standardization contributed to ensuring that all players in the health system understood what the concept of YFHS Model meant and how each one would need to participate to make a health facility youth friendly.

The approach of the innovation to improve the SRH of young people was credible and supported by international agreements. The model promotes a rights based approach for access to SRH services, responding to the resolutions of the 1997 Convention to Eliminate Discrimination Against Women. The Model also offers resources that help young people understand their sexuality, prevent unwanted pregnancies and sexually transmitted diseases (STIs), as recommended by the calls made in Cairo and Beijing.[30, 31].

The design of the innovation in Colombia specifically included ongoing participation of health representatives of the government, departments and municipalities, international agencies, civil society including young people as demonstrated by five task-force meetings between 2007 and 2008 [20] and a qualitative evaluation of the Model in 2010. This process helped shaped a Model that responded to local context of the health system and the needs of young people, increasing the likelihood of demonstrating health benefits among them [32].

The innovation was relevant to addressing the elevated rates of adolescent pregnancy by making available friendly services and information on SRH.

The YFHS Model did not meet all the characteristics of good practice recommended by the WHO-ExpandNet. The installation of the Model was neither smooth nor easy to transfer. The operation of the GSSS between private and public PHS was uneven, particularly regarding financial and human resources [33]. A documentation of the model in three regions of the department of Huila highlighted that the reasons that jeopardized the scale up of the Model were lack of funding to recruit and retain trained personnel, lack of physical spaces to implement YFHS, and disinterest in the Model among some administrative authorities [34]. The National Summative Evaluation of the Model identified that even after adopting the Model, some health facilities did not implement guidelines for serving youth [28].

#### User organization

The ExpandNet framework defines the user organization as the institution that adopts and implements the innovation on a large scale [22]. In Colombia this organization was found at three levels: a) National, b) Departmental, and c) Municipal. These three levels relied on their leadership – design and implement new policies, develop private and public PHS work plans and budgets; and commitment to scale up the Model, but the high turnover of human resources and weak political interest among some health authorities limited their implementation capacity.

- At the national level the MHSP led the implementation of the model. The MHSP had the capacity to implement it, train health personnel, and track and monitor its implementation at the departmental and municipal levels. The MHSP’s leadership and commitment strengthened its credibility; it also influenced the involvement of other institutions and sectors during the scale up process.
- At the departmental level, the RDH was responsible for implementing, providing technical assistance to municipalities and, when necessary, monitoring actions recommended in the Model as well as collecting and analysing information to be forwarded to the national level. Not all RDHs were committed to the innovation; thus the efforts and political will to scale up the Model differed. By 2013 the departments of Atlántico, Huila, Tolima, and Valle de Cauca achieved coverage of YFHS implementation per municipality higher than 90 %. In other departments coverage varied, with some as low as 5 % [29].
- At the municipal level, private and public PHS implemented the Model. Managers of social services organized and ensured provision of necessary services. Where there was commitment to the Model, there was also a smooth internal adoption of policies and the availability of health services. However, variable governance and financial resources, high turnover of trained personnel, and challenges in contracting new staff, affected the Model’s scale up and sustainability [15].

#### Resource team

The MHSP and UNFPA Colombia provided the resource team. These organizations had the leadership, credibility, commitment and ability to facilitate the implementation and national scale up of the Model.

For nearly 40 years UNFPA has worked in Colombia to build capacity for the improvement of the SRH of its population and contribute to the overall development of the country. When UNFPA signed the agreement of collaboration with the MHSP it also agreed to provide technical expertise and financial resources to launch and scale up the Model. UNFPA funded the pilot phase in 10 departments, designed and produced the manual “Friendly Health Services for Young People” and other didactic materials, and provided systems for monitoring and evaluation [20]. Similarly, UNFPA provided MHSP opportunities to establish international collaboration and to exchange experiences on scaling up YFHS with other countries. To this date UNFPA continues to provide financial support to the implementation of the Model; although the percentage of funding decreased over time (From 44 in 2007 to 27 % in 2010 and less than 5 % in 2013) [16].

The authority and leadership of the MHSP was essential for the acceptance and adoption of the Model among the different actors of the GSSS.

#### Environment

The main environmental factors, i.e., the conditions influencing the scale up process of the Model, were national policies on SRH for young people, the structure of the MHSP and GSSS, and socio-cultural perceptions about SRH services for young people as well as the ongoing effort to identify champions for the YFHS Model among government officials outside of the MHSP.

- National policies. The political commitment to improve the health of young Colombians was expressed through the formulation and implementation of various national laws and regulations. The enactment of the National Policy on SRH, the Code on Children and Adolescents, and the National Public Health Plan 2007–2010 were some of the most influential initiatives during the scale up process. The MHSP and UNFPA involved authorities from the Ministry of Education, Ministry of Information and Communication, National Learning Service, Colombian Institute for the Wellbeing of Families, as well as the Social Action and Young Colombia programmes, in order to promote and guarantee the implementation of the model within each respective agency or programme. This led to the establishment of the Inter-Sectoral National Commission to promote and guarantee SRH rights in Colombia [21]. See Table 6.
- Structure of the GSSS. The decentralized characteristics of the GSSS, the regular turnover of health authorities, and the uneven availability of and access to financial and human resources between public and private health facilities impeded the implementation and scaling up of the Model, particularly in private health facilities since they have more flexibility in choosing what services in general they offer as opposed to public ones Involvement of new RDH, LDH, and PHS authorities had to be done on an ongoing basis to ensure the continuity of the implementation of the Model.
- Socio-cultural perceptions. The demand for SRH services and information was not a common practice among young people, and was not approved by parents and other community members. Young people were uncomfortable seeking SRH services because of how they could be seen, judged, and treated. Among adults, the beliefs and perceptions about the SRH of young people, including early initiation of sexual intercourse, contraception and pregnancy in this group were seen as incompatible to their religious beliefs and social practices [28]. Thus, the inclusion of young people, parents and community members as stakeholders was critical for the adoption and scale up of the model.

The first link with stakeholders came from the formation of “social oversight committees of young people”, known as “Veedurías” in Spanish. “Veedurías” were formed by young people, parents, and other community members. They disseminated information about the characteristics of YFHS to the general public. They also participated in the design and monitoring of interventions in health facilities adopting the model. Until 2012, 38 active “veedurías” supported the scale up the model in 11 departments of the country [35].

The second link with stakeholders was with health personnel who recognized young people as individuals with rights to information about SRH and access to quality SRH services. Their high turnover demanded continuous sensitization and training on youth friendly services. The MHSP provided this training and coordinated with ten universities across the country to establish a Certificate of Reproductive Rights and Sexual Health of Adolescents. Between 2012 and 2013, 302 health personnel graduated from the programme and were qualified to work with young people [35].

#### Scaling up strategy

According to the WHO-ExpandNet framework the scaling up strategy can be: Vertical, horizontal, diversified, and spontaneous. Colombia adopted a combination of vertical and horizontal strategies.

The vertical strategy consisted of the institutionalization of the Model within national health public policies and its inclusion in the Mandatory Health Plan of the GSSS. To ensure that the scale up of the YHFS maintained its quality standards, the MHSP -through Act. 1122 and Resolution 425, mandated its implementation. At the sub-national levels, public and private PHS adopted the Model based on its context and capabilities. This horizontal strategy consisted on providing on-going support and training on YFHS to different members of the GSSS.

### Strategic management of the scaling up process

#### Dissemination and advocacy

The basis of the advocacy effort was MHSP’s commitment to making YFHS available nationally. The enactment of 1122 Act recognized the Model as an element of a multifaceted strategy to improve the SRH of young people. The Resolution 425 or Collective Intervention Plan strengthened the adoption of the model by different actors of the GSSS. Likewise, the MHSP contributed to international agreements and inter-institutional cooperation that supported this process. In 2011 the President of Colombia led the initiative to sign the Commitment of the political parties and movements to promote and guarantee sexual and reproductive health rights. Through this agreement government authorities were accountable for the execution and continuity of interventions that seek to reduce the rate of adolescent pregnancy, including the Model [21].

Advocacy among community members was also essential to achieve acceptance and adoption of the model. This was obtained, among other strategies, by establishing the “veedurías.”

#### Organizational process

The scale up process of the model was in phases, consistent, and continuous. Each year the number of regions executing the model, PHS adopting the Model, conducting training workshops and training personnel increased progressively.

The MHSP was consistent in running the processes of dissemination, training, and technical assistance during the scale up of the Model. It was also flexible to adapt it according to local characteristics and needs as well as the infrastructure and availability of public and private health institutions. For instance, in 2013 from a total of 817 health facilities implementing the Model the majority adopted the differentiated service modality, and to a lesser extent, the friendly unit and friendly centre modalities [29].

The scale up of the Model was decentralized to departments with variations in speed, scope of the population, and places to be reached. For example, a review of the implementation of the model in Huilla details how this department managed to expand the Model in 20 municipalities within 2 years [34]. By 2013, the scale up of the model reached 30 of the 32 departments. Similarly, between 2009 and 2013, Atlántico and Valle del Cauca reached 100 % coverage of YFHS per municipality. On the contrary, other departments were slower, abandoned the initiative, or did not implement it at all due to a weak political commitment among town counsellors, health officials or hospitals’ managers thus less financial and human resources [29, 36].

The demographic and geographic limitation of scaling up the Model was directly related to the scope of the PHS. According to the GSSS, some private PHS serve the population mainly on a contributory scheme, whereas public PHS usually assist the uninsured or those who cannot afford medical care. Coverage was lower among private PHS because not all of them adopted the Model, and in the ones who did it young people had to use their insurance or pay to get the services [25].

#### Costs/resource mobilization

The MHSP, UNFPA, UNICEF, and the Andean Plan to Prevent Adolescent Pregnancy were the main sources of funding for the scale up effort. Allocated resources were adequate and sufficient to carry out the pilot phase, train health personnel, establish new YFHS as well as processes for monitoring and evaluation, develop and distribute educational materials, and organize interagency events to discuss the progress of the model.

The scale up of the Model, however, did not have reliable and predictable long-term funding at the municipal level. Reviews of the Model indicate that delays in allocating financial resources from the national level to PHS limited the availability of supplies and medicines for patients. PHS that did not incorporate the model in their institutional Development Plan were not able to guarantee continuous funding of trained personnel and commitment to sustain the model [15, 33, 34].

#### Monitoring and evaluation

The MHSP designed robust monitoring and evaluation processes with clear data collection tools. The MHSP also assigned specific institutions and personnel to collect, analyse, and submit that data [20]. Table 7 provides the list of instruments used to measure the quality and coverage of the YFHS Model.

**Table 7:** Measurement tools to assess the quality and coverage of the AFHS Model

• → Vital statistics DANE (Births and deaths)	
• → Individual registry of service provision (RIPS in Spanish)	
• → Single registry of affiliates to the social protection system (RUAF in Spanish)	
• → Management system indicators	
• → Annex A4 – Tool to analyse health services based on the components of the AFHS model	
• → Annex A11 – Self-administered survey for adolescents and young people	

Source: Ministerio de Salud y Protección Social Colombia, UNFPA. (2007). Servicios amigables en salud para adolescentes y jóvenes. Un modelo para adecuar las respuestas de los servicios de salud a las necesidades de adolescentes y jóvenes de Colombia. Bogotá D.C., Colombia

**Fig. 1:**
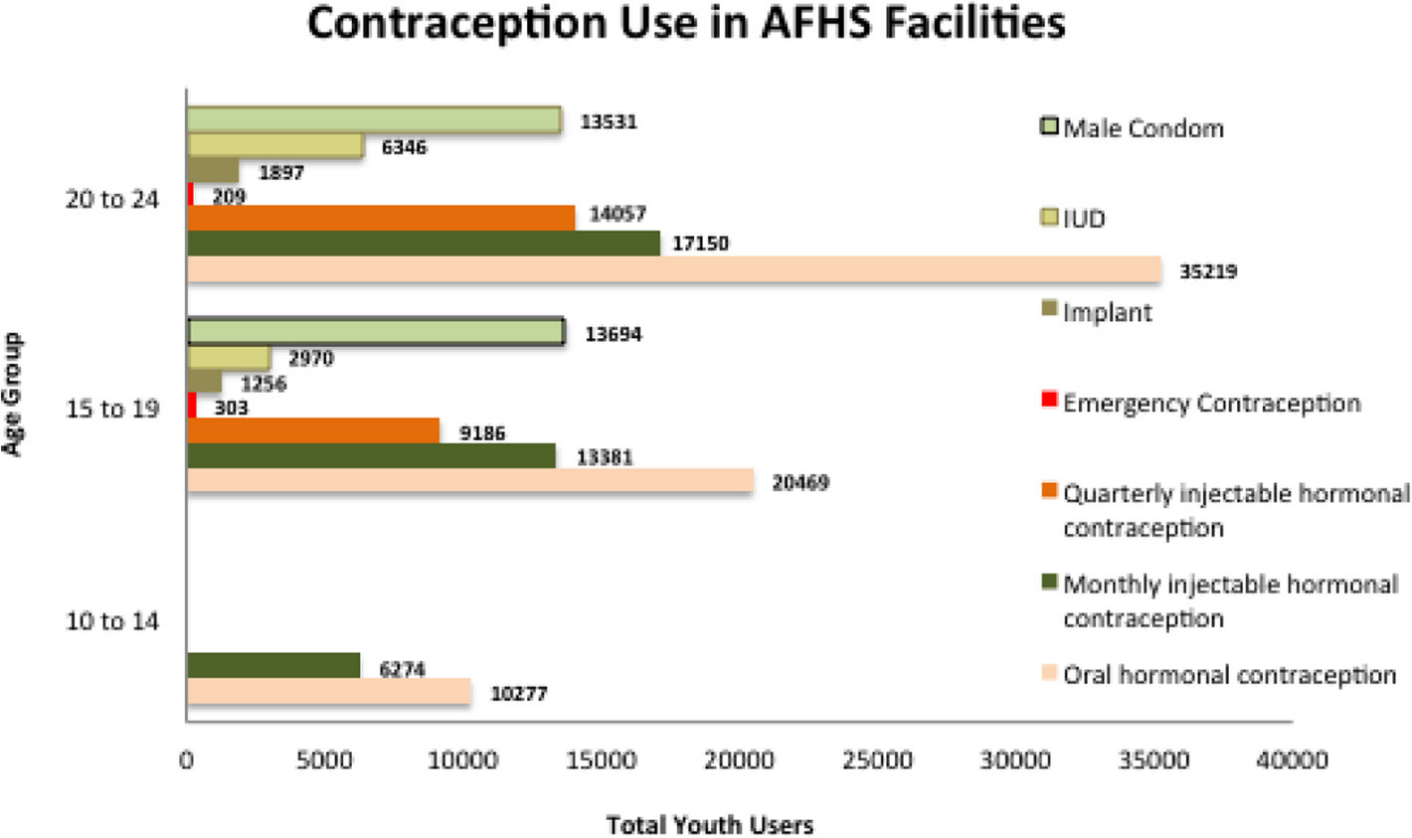
Contraception used in AFHS facilities. Source: Colombia MSPS: Consolidado de Indicadores 2013. Servicios de Salud Amigable para Adolescentes y Jóvenes. 2014

**Fig. 2:**
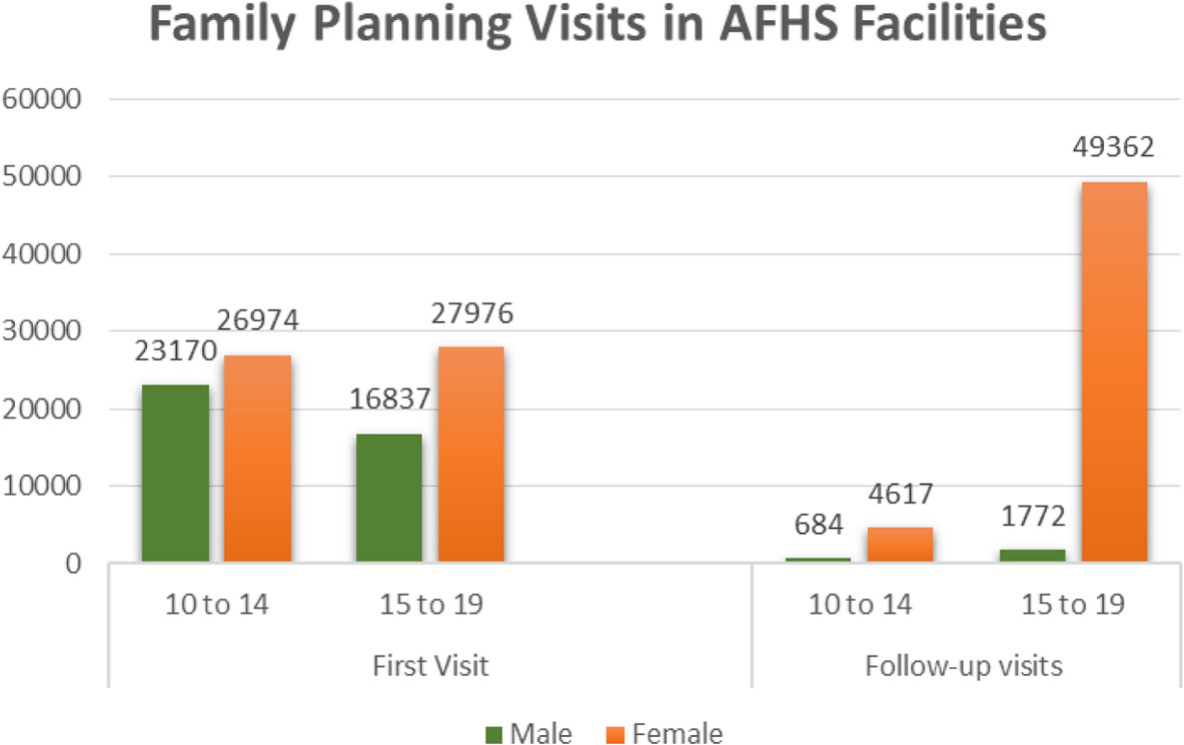
Family planning visits in AFhs facilities. Source: Colombia MSPS: Consolidado de Indicadores 2013. Servicios de Salud Amigable para Adolescentes y Jóvenes. 2014

This process was in place since the beginning of the model. In 2009 and 2010, the MHSP carried out surveys to track implementation of the model (Annex A4 and Annex A11) [37]. The results enabled the MHSP to identify the determinants of success and failure of the scale up process; as well as criteria to point to which components of the model required strengthening or further attention to improve the quality of services provided. Table 8 and Figs. 1 and 2 summarize the findings of the evaluation.

**Table 8:** Summary of the 2010 summative evaluation of the AFHS Model

Total of AFHS	733
Number of facilities reporting implementation of the AFHS Model	372
Total of health professionals implementing the AFHS Model	43.605
Total of youth visits	280.564
Total of family planning visits	151.168
Total of youth using contraception	403.804
Total of youth seeking SRH advisory services	140.954
Total of youth deliveries	10.683

Source: Colombia MSPS: Consolidado de Indicadores 2013. Servicios de Salud Amigable para Adolescentes y Jóvenes. 2014

In practice, the information management system was inadequate at different levels of the user organization, especially on making data available on time [15, 37]. Getting immediate, reliable and updated data about the scale up of the model remains a challenge due to the continuous turnover of data analysts.

#### Building on the lessons of the effort to scale up YFHS and taking remedial action

The implementation cycle of the YFHS Model was characterized by ongoing identification of performance components that needed improvement and taking of remedial actions. In 2009, the MHSP organized the first national meeting on lessons learned from the implementation of the Model with the participation of representatives of PHS implementing the Model and young people.

The meeting concluded with a list of suggestions to improve the scope of the Model as the scale up occurred, which became the basis for the MHSP’s further strategic remedial action. Overall it took almost three years to document the accomplishment of those actions. The MHSP took seriously the need to strengthen youth participation in the implementation of the Model. Since 2012, the MHSP implemented the idea of “veedurías” (social oversight committees) for young people within the Model [38] and identified and strengthened youth networks and organizations as well as created regional and national youth councils to prevent adolescent pregnancy [39]. In 2013 the MHSP established an agreement that funded youth strategies that would enhance public participation to identify local actions to prevent adolescent pregnancy, promote SRH rights, and prevent STIs including HIV [40].

Another remedial action was the development of training strategies for healthcare providers on youth SRH and rights. The MHSP partnered with UNFPA and national universities to create a graduate curricula on adolescent SRH, which as mentioned in previous paragraphs, reached over 300 health providers in four major cities [41].

The MHSP has also worked on adapting the Model for young people coming from indigenous groups. By early 2014 it had been adapted by the indigenous communities of Nariño and la Guajira [42].

Finally, the MHSP has looked for strategies to strengthen the procurement processes of YFHS and standardize the tools used for data collection. To do so, it developed a standard matrix on Microsoft Excel that included YFHS indicators, definitions, and guidelines to facilitate the introduction of YFHS data. As this matrix was distributed nationally, the MHSP trained health providers on its use. Despite these efforts, the use of this matrix and the availability of national YFHS coverage data remains a challenge.

## Discussion

The efforts to establish and scale-up SRH services for young people in Colombia have led to significant achievements but have also faced a range of challenges. Despite this, Colombia is one of few LA countries that has achieved national implementation of YFHS although with an uneven coverage across the departments. The lessons learned from this effort could help strengthen the work in Colombia, and as well as in other countries are doing to provide comprehensive SRH services to young people in a sustainable manner.

What are the elements that made possible the implementation and scale up of the YFHS Model in Colombia? The WHO-ExpandNet framework allowed us to identify five elements that contributed to this process.

First, the Model was built on momentum in the country to support initiatives that improve the SRH of prevent pregnancies among young people. National health policies established clear guidelines for the implementation of the Model within the GSSS. Clear policies and implementation guidelines on the YFHS Model was an important attribute in the scale up process.

Second, political stakeholders and advocates strongly endorsed the YFHS Model. The leadership of the MHSP and UNFPA helped ensure the recognition of the Model with relevant government ministries, and within the health system. At the same time the MHSP built significant alliances with international institutions. This solid inter-sectoral and interagency collaboration was important in providing the technical and financial resources necessary as the scale up process occurred.

Third, the work of the MHSP and UNFPA as the resource team, and the former as a user organization as well, favoured the scale up process by providing the necessary technical and financial resources.

Fourth, the continuous involvement of different members of the community and young people themselves in the improvement of health facilities led to ownership and sustainability of these services, which helped address negative socio-cultural perceptions about the provision of SRH information and services to young people. The strong involvement and community empowerment was key to reach large-scale implementation.

Fifth, despite its limitation, the presence of a monitoring and evaluation component allowed the identification of elements in the model that required strengthening or further attention. Effective use of information contributed to the continuous improvement of the model as the scale up occurred.

The scaling up of the model did not follow a smooth process. The elements that limited or slowed down this effort were: First, the insufficient number of health personnel trained in youth health and their high turnover. Second, a decentralized SGSS that caused variability in institutional management and distribution of financial resources at the departmental and municipal levels. Given that funding was dependant on the inclusion of the Model in the municipal operating plan, decentralization did not guarantee continuation of health personnel implementing the Model. Finally, the negative perceptions among community members to provide SRH information and services to adolescents hindered implementation.

So far no attempt has been made to measure the impact of the implementation of the Model in terms of uptake of services or health outcomes. The development of a monitoring and evaluation framework is under way and this will hopefully provide data about the degree to which the Model succeeds in helping young people protect their SRH. Although more than 800 clinics have become youth friendly, which in itself is a major achievement, there is still a long way to go to reach the majority of adolescents in the country. Both vertical and horizontal scale-up must be pursued to reach a larger number of young people.

## Conclusion

The experience of scaling up youth friendly health services in Colombia demonstrates the need to establish clear policies and implementation guidelines at all levels of the health system. Inter-sectoral and interagency collaboration to establish the standard of required quality of health care and the identification of champions that lead and strengthen the implementation of the YFHS Model facilitate its implementation and monitoring. It also illustrates the importance of emphasizing the quality of services and continuous training of health personnel in the SRH of young people, as well as the inclusion of young people in the design, execution, and monitoring of services.

The main challenges in scaling up the YFHS Model in Colombia were the decentralized management system and the lack of commitment among some of regional and local authorities that limited the continuity, sustainability and increase in coverage of the model. It highlights the need to invest time and resources right from the beginning to get the buy-in and support of the lower levels, and ensuring their participation.

## Footnotes

1 The World Health Organization (WHO) defines young people as those between the ages 10–24. It includes Adolescents (10–19) and youth (15–24).
